# The Danger of the Vaginal Injection

**Published:** 1897-09

**Authors:** 


### THE DANGER OF THE VAGINAL INJECTION.

The case reported by Dr. Wells at a recent meeting of the Society of Alumni of the Charity Hospital—for by its old name we still prefer to call the hospital—which we print elsewhere in this issue, may well serve as the text for renewed cautionary remarks as to the danger of the self-administered vaginal injection. In this case a woman who had had a natural labor seventeen days before, and was at the time in a perfectly normal condition, save for a vaginal discharge observed by herself and a state of retroversion of the uterus discovered shortly afterward by Dr. Wells, took it into her head to administer to herself a vaginal douche. Accordingly, she filled a Davidson syringe with plain water, thrust the long, rectal nozzle of the instrument into her vagina to the whole length, and squeezed the bulb forcibly. She was at once seized with severe abdominal pain and came very near going into a state of collapse. Having escaped the immediate danger, she suffered from a continuance of the pain, which extended up to the region of the diaphragm and was accompanied with tympanitic distention of the abdomen and a condition highly indicative of general peritonitis. Fortunately, the trouble was soon narrowed down to an inflammatory process seated in the pelvis and giving rise to a massive exudate—a state of things bad enough, to be sure, and tolerably certain to trouble her more or less for years to come, perhaps seriously, but not putting her life in immediate jeopardy. For a time Dr. Wells may well have been in doubt, as he says he was, as to whether he ought not to resort to abdominal section.

It seems quite probable, as Dr. Wells intimates, in view of the retroverted state of the uterus in this case, that the nozzle of the syringe was thrust into the uterine canal, possibly nearly to the fundus of the organ, and septic or at least irritating material forced through a Fallopian tube into the peritoneal cavity. It is stated in the account that the woman had had gonorrhœa some years before. The entrance of the nozzle into the uterus, perhaps carrying with it some infectious matter from the vagina, and certainly involving a flooding of the uterine cavity with water that in all probability was not free from septic material, furnishes an obvious and plausible explanation of the manner in which the pelvic inflammation was set up in the case under consideration, but such an occurrence does not, by any means, constitute the sole mishap of a vaginal injection improperly administered. In order that the injection may be forced into the uterus and through a Fallopian tube, it is not in the least necessary that the nozzle should enter the uterine canal, that one of its apertures should be so situated as to send a stream into the uterus, or that the organ should be retroverted; the one indispensable condition of safety is the provision of a free passage for the outflow of the fluid injected.

If a vaginal douche is to be employed during the puerperal period, says Dr. Wells, it should be given only by the physician or a thoroughly competent nurse. That is perfectly true, but, apart from the puerperal condition, danger lurks in a vaginal injection administered without due precaution. How does the uninstructed woman usually proceed when she sets about giving herself a vaginal injection? She squats on some convenient utensil, thrusts in the nozzle, which is almost always of a kind utterly unfit for the purpose, and pumps rapidly, if the syringe is of the bulb type, so as to be over with the unpleasant affair as soon as may be, or hangs the bag high, if she is using an apparatus of the fountain type. In either case, if she has the good luck not to drive the nozzle into her uterus—and there is many a woman going about in tolerable health, so far as she knows, whose uterine orifice will readily admit an ordinary syringe nozzle—in either case, we repeat, she rather suddenly fills her vagina to repletion, even to distention, perhaps with a liquid that, whether by reason of its temperature or of something it holds in solution, may act as a stimulus to spasmodic contraction of the pelvic and perineal muscles. Fortunate is it for her if her vulvo-vaginal sphincter, although never designed to grasp so tiny an object, does not grip the nozzle so vigorously as to imprison the water under a pressure that is very apt to force it into the uterus. It is necessary very often to intrust women with the self-administration of vaginal injections—not, of course, in the lying-in period—and they should always be instructed how to guard against their dangers, and in particular they should be provided with a nozzle so constructed that no amount of contraction about its shank can close the vaginal outlet. Unfortunately, not one nozzle in a thousand is so constructed. —*Editorial*, *New York Medical Journal.*

